# Epidemiology and Consequences of Drinking and Driving

**Published:** 2003

**Authors:** Ralph Hingson, Michael Winter

**Affiliations:** Ralph Hingson, Sc.D., is a professor in the Social and Behavioral Sciences Department and associate dean for research, and Michael Winter, M.P.H., is a data analyst in the Data Coordinating Center, both at the Boston University School of Public Health, Boston, Massachusetts

**Keywords:** drinking and driving, epidemiological indicators, AODR (alcohol and other drug related) accident mortality, traffic accident, impaired driver, risk factors, BAC, seat belt, driver performance, license control, age of AODU (alcohol and other drug use) onset, law enforcement, trend, deterrence of AODU, gender differences, racial differences, age differences

## Abstract

Alcohol is a major factor in traffic crashes, and crashes involving alcohol are more likely to result in injuries and deaths than crashes where alcohol is not a factor. Increasing blood alcohol concentrations (BACs) have been linked to increased crash risk. Male drivers, particularly those ages 22 to 45; people with drinking problems and prior drinking and driving convictions; and drivers who do not wear safety belts are disproportionately likely to be involved in alcohol-related fatal crashes. Alcohol-dependent people are over-represented in all alcohol-related traffic crashes, as are those who begin drinking at younger ages. Though there are more than 82 million drinking–driving trips in a given year at BACs of 0.08 percent and higher (and 10 percent of drinking–driving trips are at BACs of 0.08 percent and higher), there are only 1.5 million arrests for drinking and driving each year. Despite overall marked reductions in alcohol-related traffic deaths since the early 1980s, there has been little reduction since the mid-1990s, and alcohol-related traffic deaths have increased slightly in the past 3 years.

Despite reductions in alcohol-related traffic fatalities since the early 1980s, alcohol remained a factor in 41 percent of the traffic deaths recorded in the United States in 2002. This article examines the epidemiology of alcohol-related crashes, injuries, and deaths; characteristics of alcohol-related fatalities, fatal crashes, and drivers in alcohol-related fatal crashes; alcohol dependence and alcohol-related crashes; survey data on self-reported drinking and driving; and trends in drinking and driving.

## Traffic Crash Deaths and Injuries

Traffic crashes are the leading cause of death in the United States for people ages 2 to 33 (National Highway Traffic Safety Administration [NHTSA] 2003*b*). According to NHTSA, 41 percent of people fatally injured in traffic crashes were in alcohol-related crashes (i.e., those in which a driver or pedestrian had a blood alcohol concentration [BAC] greater than zero), and 35 percent were in crashes involving someone with a BAC of 0.08 percent or higher. Of the total number of people injured in traffic crashes, 9 percent were injured in alcohol-related crashes (225,000 out of 2,926,000). (For more information on BAC and fatal crashes, see the sidebar on p. 66.)

**BAC and Fatal Crash Involvement**

The proportion of alcohol to blood in the body is referred to as the blood alcohol concentration (BAC). A person’s BAC is determined by his or her drinking rate and by the body’s absorption, distribution, and metabolism of the alcohol. What follows is a brief description of how these processes affect BAC measurement and the consequences of BACs for driving.

***Absorption and Distribution***

When alcohol is consumed, it passes from the stomach and intestines and is absorbed into the bloodstream. As it circulates in the bloodstream, alcohol distributes itself evenly throughout all the water in the body’s tissues and fluids.

Because of the way alcohol distributes itself throughout body fluids, it is possible to measure a person’s alcohol level by testing the urine, saliva, or water vapor in the breath, as well as by testing the blood. Law enforcement agencies primarily use breath testing, but they often convert breath-test results to equivalent blood alcohol measurements, because early drunk driving laws based their limits on blood tests (National Highway Traffic Safety Administration [NHTSA] 1990). In cases of alcohol-related traffic fatalities, however, blood testing must be used to estimate alcohol levels.

In the United States, blood alcohol measurements are based on the amount of alcohol, by weight, in a set volume of blood. For example, a BAC of 0.10 percent—a level at which it is illegal to drive in the United States—is the equivalent of 0.10 grams of alcohol per 100 milliliters of blood. This translates, by weight, to a proportion of just under 1 gram of alcohol for every 1,000 grams of blood in the body (Jones and Pounder 1998).

***Breakdown in the Body***

Within a few seconds after ingestion, alcohol reaches the liver, which begins to break it down, or metabolize it. Any BAC measurement therefore reflects not only a person’s drinking rate but also his or her rate of metabolism.

**Table:** 

Driver’s blood alcohol concentration (BAC) in this range:	Multiplies the chances of being killed in a single-vehicle crash increase by:						

	Ages	For males			For females		
		16–20	21–34	35+	16–20	21–34	35+
0.02–0.049		5	3	3	3	3	3
0.05–0.079		17	7	6	7	7	6
0.08–0.099		52	13	11	15	13	11
0.10–0.149		241	37	29	43	37	29
0.15+		15,560	572	382	738	572	382

SOURCE: Zador et al. 2000.

The human body metabolizes alcohol much more slowly than it absorbs alcohol, so the concentration in the body increases when the person consumes additional drinks before earlier drinks have been metabolized.

Factors that influence BAC during and after drinking a given amount of alcohol include age, gender, the proportion of body mass made up by fatty tissue, and whether food is eaten with the alcoholic beverage. Although individual rates can vary, on average, a 170-pound man who has four drinks in an hour on an empty stomach, or a 135-pound woman who has three drinks under similar conditions, would reach a BAC of 0.08 percent (NHTSA 1992).

***Consequence: Crash Risk***

In 2002, 84 percent of the drivers who had been drinking and were involved in fatal crashes had BACs at or above 0.08 percent (NHTSA 2003). Most States have established a BAC of 0.08 percent as the legal level of intoxication.

A number of studies have identified how the physiologic responses needed for safe driving are impaired beginning at BACs of 0.08 percent or lower. Experimental laboratory studies have reported on the physical deficits experienced with a 0.08 percent BAC, a level reached by a 170-pound man after consuming four drinks in 1 hour on an empty stomach, or by a 135-pound woman consuming three drinks. These deficits include:

- Reduced peripheral vision
- Poorer recovery from glare
- Poor performance in complex visual tracking
- Reduced divided attention performance (i.e., the simultaneous performance of two or more tasks such as tracking, visual search, number monitoring, and detection of auditory stimuli) (Moskowitz and Fiorentino 2000).

Driver simulation and road course studies have revealed poorer parking performance, poorer driver performance at slow speeds, and steering inaccuracy at BACs of 0.05 percent and higher (Finnigan and Hammersley 1992; Hindmarch et al. 1992; Starmer 1989). Roadside observational studies have identified increased deterioration of speeding and breaking performance (Damkot et al. 1975).

Finally, in a comparison of alcohol test results, Zador (1991) found that each 0.02 increase in a driver’s BAC nearly doubled his or her risk of being in a single-vehicle fatal crash. This study examined alcohol test results of drivers killed in single-vehicle fatal crashes and compared these results with breath alcohol samples provided by 2,850 drivers stopped in the same States as part of a national roadside survey of drivers. To match driver fatalities to the roadside breath-testing exposure, the crash times, days, and roadway types were restricted to those used in the survey of drivers. The study found that, for all age and gender groupings, the likelihood of being a fatally injured driver was at least 9 times greater at BACs of 0.05 to 0.09 percent than at zero BAC. For each 0.02 percent increase in BAC, the fatal crash risk increased even more for drivers under age 21 and for female drivers.

This study was recently updated (Zador et al. 2000) (see table). Alcohol test results from drivers stopped in the 1996 National Roadside Survey of weekend nighttime drivers were compared with the alcohol involvement of drivers in weekend nighttime single-vehicle fatal crashes, as determined by NHTSA for 1995 and 1996. Relative to nondrinking drivers, drivers in all age and gender groups examined who had BACs between 0.08 percent and 0.099 percent had at least an 11 times greater risk of dying in a single-vehicle crash. Male drivers age 16 to 20 with 0.08 percent BAC had 52 times greater risk than zero-BAC drivers of the same age.

In addition, a recent review of 112 studies provided strong evidence that impairment in driving skills begins with any departure from zero BAC (Moskowitz and Fiorentino 2000). The majority of studies reported impairment by 0.05 percent BAC. The authors concluded that virtually all drivers tested in the studies reviewed exhibited impairment on some critical driving measure by the time they reached a BAC of 0.08 percent.

Evidence regarding driving impairment at BACs of 0.08 percent and higher—as well as evidence from a series of studies examining the effects of lowering legal blood alcohol limits from 0.10 percent to 0.08 percent—prompted Congress in October 2000 to pass legislation to withhold Federal highway construction funds from States that do not adopt 0.08 percent as the per se legal BAC. Currently 44 States have adopted 0.08 percent as the legal limit. A recent review of 9 studies examining the first 16 States to lower the legal limit from 0.10 percent to 0.08 percent found that the median decline in alcohol-related motor vehicle fatalities following the adoption of the 0.08 law was 7 percent, providing “strong” (Shults et al. 2001, page 71) evidence that 0.08-percent BAC laws are effective in reducing alcohol-related crash fatalities (Shults et al. 2001).

—Ralph Hingson and Michael Winter

ReferencesDamkotDKPerrineMWWhitmoreDGOn the Road: Driving Behavior and Breath Alcohol ConcentrationVolumes I and II (Technical Report). Pub. No. DOT HS–364–37567Washington, DCU.S. Department of Transportation1975FinniganFHammersleyRThe effects of alcohol on performanceSmithAPJonesDMHandbook of Human Performance. Vol. 2. Health and PerformanceLondonAcademic Press199273126HindmarchIBhattiJZStarmerGAThe effects of alcohol on the cognitive function of males and females and on skills relating to car drivingHuman Psychopharmacology: Clinical and Experimental721051141992JonesAWPounderDJMeasuring blood-alcohol concentration for clinical and forensic purposesKarchSBDrug Abuse HandbookBoca Raton, FLCRC Press1998327356MoskowitzHFiorentinoDA Review of the Literature on the Effects of Low Doses of Alcohol on Driving Related SkillsPub. No. DOT HS–809–028Springfield, VAU.S. Department of Transportation, National Highway Traffic Safety Administration2000National Highway Traffic Safety Administration (NHTSA)Alcohol and Highway Safety 1989: A Review of the State of KnowledgePub. No. DOT HS–807–557Washington, DCU.S. Department of Transportation1990National Highway Traffic Safety Administration (NHTSA)*BAC Estimator* [computer program]Springfield, VANational Technical Information Service1992National Highway Traffic Safety Administration (NHTSA)Traffic Safety Facts 2002: AlcoholPub. No. DOT HS–809–606Washington, DCU.S. Department of Transportation2003ShultsRElderRSleetDReviews of evidence regarding interventions to reduce alcohol-impaired drivingAmerican Journal of Preventive Medicine21Suppl 46688200110.1016/s0749-3797(01)00381-611691562StarmerGAEffects of low to moderate doses of ethanol on human driving-related performanceCrowKEBattRDHuman Metabolism of Alcohol. Vol. I. Pharmacokinetics, Medicolegal Aspects, and General InterestsBoca Raton, FLCRC Press1989101130ZadorPLAlcohol-related relative risk of fatal driver injuries in relation to driver age and sexJournal of Studies on Alcohol5243023101991187570110.15288/jsa.1991.52.302ZadorPKrawchukSVoasRAlcohol-related relative risk of driving fatalities and driver impairment in fatal crashes in relation to driver age and gender: An update using 1996 dataJournal of Studies on Alcohol6138739520001080720910.15288/jsa.2000.61.387

Traffic crashes are more likely to result in death or injury if alcohol is involved. Of all alcohol-related crashes in 2002, 4 percent resulted in a death, and 42 percent in an injury. In contrast, of the crashes that did not involve alcohol, 0.6 percent resulted in a death, and 31 percent in an injury.

Many people other than drinking drivers are killed in crashes involving drinking drivers. Overall in 2002, 44 percent of those who died in traffic crashes involving a drinking driver with a BAC of 0.01 percent or higher were people other than the drinking driver: 7 percent were other drivers in vehicles struck by drinking drivers, 22 percent were passengers in vehicles with drinking drivers or struck by drinking drivers, 13 percent were pedestrians, and 2 percent were bicyclists. In 2002, 573 children younger than age 16 died in crashes involving drinking drivers.

## Characteristics of Alcohol-Related Fatalities and Fatal Crashes

Data from the Fatality Analysis Reporting System (FARS) (NHTSA 2003*a*) reveal that alcohol involvement in fatal crashes varies considerably by gender, age, race/ethnicity, type of vehicle driven, time of day, day of the week, and whether the person involved was a driver, motor vehicle passenger, or pedestrian.

### Characteristics of Alcohol-Related Fatalities

#### Gender

Males are more likely than females to be involved in alcohol-related fatal crashes. In 2002, 78 percent of people killed in alcohol-related crashes (including drivers, passengers, and pedestrians) were male. Forty-six percent of male traffic deaths are alcohol related, compared with 29 percent of female traffic deaths.

#### Age

Traffic deaths among elderly people and children are less likely to be alcohol related than those among young and middle-aged adults. As shown in table 1, only 15 percent of traffic deaths among adults age 65 and over were alcohol related, compared with 23 percent of traffic deaths among children under age 16, 37 percent among 16- to 20-year-olds, 57 percent among 21- to 29-year-olds, 53 percent among 30- to 45-year-olds, and 38 percent among 46- to 64-year-olds. Alcohol-related traffic deaths are more likely to occur at lower BACs among 16- to 20-year-olds, compared with other age groups. A majority of alcohol-related traffic deaths among 16- to 20-year-olds occur at below 0.15 percent BAC (i.e., referring to the highest BAC of a driver or pedestrian involved in the crash). Overall, however, a majority of traffic deaths occur at above 0.15 percent BAC (NHTSA 2003*a*).

#### Race/Ethnicity

The FARS does not routinely record the race and ethnicity of people who die in motor vehicle crashes. However, from 1990 to 1994 a special initiative linked nearly 200,000 records from FARS with death certificate information on race and ethnicity from the National Bureau of Health Statistics (Voas and Tippetts 1999). Information was available only for people who died in crashes, not drivers who survived fatal crashes. During that time period, 72 percent of people killed in alcohol-related fatal crashes were White, 12.1 percent were African American, 2.4 percent were Native American, 1.2 percent were Asian Americans and Pacific Islanders (AAPIs), and 12.7 percent were Hispanic (including Mexican Americans [8.7 percent], Puerto Ricans [0.6 percent], Cubans [0.3 percent], Central and South Americans [1.1 percent], and people of other Hispanic origins [2.0 percent]). During the same period, according to the U.S. Census Bureau, 83 percent of the U.S. population was White, 13 percent was African American, 1 percent was Native American, 3 percent was AAPI, and 10 percent was Hispanic.

The proportion of traffic fatalities that were alcohol related varied considerably by race and ethnicity. It should be noted that all alcohol-related traffic deaths are not represented here because the data set does not include traffic deaths from crashes in which the drinking driver survived. Among all groups, 38 percent of traffic deaths were alcohol related. Native Americans had the highest percentage of traffic deaths that were alcohol related (68 percent). Whites and African Americans had similar proportions (38 percent and 39 percent, respectively). Within Hispanic groups there was considerable variability: Alcohol was involved in 50 percent of traffic deaths among Mexican Americans, 42 percent among Central/South Americans, 36 percent among Puerto Ricans, and 24 percent among Cubans. AAPIs had the lowest percentage of alcohol-related traffic deaths of any ethnic group (19 percent).

In every racial or ethnic group examined, a higher proportion of male than female deaths were alcohol related. This was true for drivers, passengers, pedestrians, and cyclists. In almost every racial/ethnic group, the age group with the highest percentage of drivers and pedestrians who died in alcohol-related crashes was the 21- to 49-year-old group (Voas and Tippetts 1999).

#### Crash Participants and Vehicles

The percentage of traffic deaths that are alcohol related also varies depending on the role of the person killed in the crash (i.e., whether the person killed was the driver, passenger, or pedestrian) and by the type of vehicle involved. In 2002, 41 percent of the drivers killed in crashes were killed in alcohol-related crashes, compared with 37 percent of passenger deaths and 47 percent of pedestrian deaths. Of all pedestrian deaths, 17 percent involved a driver who had been drinking and 38 percent involved a pedestrian who had been drinking. In 7 percent of pedestrian deaths, both the driver and the pedestrian had been drinking (NHTSA 2003*f* ).

In 2002, 39 percent of deaths of motorists in automobiles were alcohol related (7,954 out of 20,416), compared with 42 percent of deaths of motorists in vans or light trucks (5,148/12,182), and 44 percent of motorcycle deaths (1,422/3,244). Though deaths of bicyclists (from both crashes with cars and bike accidents not involving cars) are much less frequent, 37 percent of them in 2002 were alcohol related (i.e., either the driver or the bicyclist was drinking) (243/660). In contrast, only 13 percent of deaths among motorists in large trucks were alcohol related (87/684) (NHTSA 2003*a*).

### Characteristics of Alcohol-Related Fatal Crashes

As shown in tables 2 and 3, alcohol-related traffic crashes are more likely to occur at night and on weekends. Seventy-seven percent of fatal alcohol-related traffic crashes occurred between 6:00 p.m. and 6:00 a.m., compared with 33 percent of non-alcohol-related fatal crashes (NHTSA 2003*a*). More alcohol-related fatal crashes occur on Saturday (24 percent) than any other day, followed by Sunday (21 percent) and Friday (16 percent).

## Drivers in Alcohol- Related Fatal Crashes

The FARS data also provide information on the characteristics of drivers involved in alcohol-related fatal crashes—their age, gender, previous convictions and license suspensions, BAC, and safety belt use.

### Age and Gender

Drivers between the ages of 16 and 20, and especially those ages 21 to 45, are likely to be involved in alcohol-related fatal crashes at a rate that is out of proportion to their percentage of the population. Although 14 percent of drivers in alcohol-related fatal crashes in 2002 were between 16 and 20, this age group represents only 7 percent of the population. Likewise, 49 percent of drivers in alcohol-related fatal crashes were ages 21 to 45, and this age group makes up 35 percent of the population. Most drivers in alcohol-related fatal crashes are male (73 percent) (NHTSA 2003*a*,*e*).

### License Suspension and Drinking and Driving Conviction

Drivers in fatal crashes who had positive BACs were more likely than other drivers in fatal crashes to have had their driver’s license suspended. Eight percent of drivers in fatal crashes who had BACs of zero had a suspended license, compared with 19 percent of drivers with BACs between 0.01 and 0.07 percent, 21 percent of drivers with BACs between 0.08 and 0.14 percent, and 24 percent of drivers with BACs of at least 0.15 percent.

Only a small fraction of drivers in fatal crashes had drinking and driving convictions in the previous 3 years (3.2 percent) (see table 4). But of the drivers in alcohol-related fatal crashes, 8.4 percent had prior drinking and driving convictions. Further, the higher the BAC of drivers in fatal crashes, the greater their likelihood of a prior conviction (NHTSA 2003*a*).

Three States have recently established lower legal BACs for drivers with prior drinking and driving convictions, and research indicates that such laws reduce the proportion of fatal crashes that involve drivers with prior convictions, particularly fatal crashes in which the driver had a BAC of 0.15 percent or higher (Hingson et al. 1998).

### Driver BAC and Crash Characteristics

The higher the BAC of a driver in a fatal crash, the greater the likelihood that the crash involved only one vehicle. Thirty percent of zero-BAC drivers in fatal crashes were involved in single-vehicle crashes, compared with 68 percent of drivers with BACs of 0.15 percent or higher.

The BACs of drivers in fatal crashes were also related to driving behaviors that contributed to the fatal crash (see table 5). For example, only 23 percent of zero-BAC drivers in fatal crashes failed to keep in their lane or ran off the road, compared with 58 percent of drivers with BACs of 0.15 percent or higher.

The most harmful event in fatal crashes also varied considerably according to driver BAC (see table 6). Among drivers in fatal crashes who had a zero BAC, only 10 percent experienced a vehicle overturn and only 10 percent struck a fixed object. In contrast, among drivers with BACs of 0.15 percent or higher, 28 percent experienced a vehicle overturn and 33 percent struck a fixed object (NHTSA 2003*a*).

### Speeding

NHTSA considers a fatal crash to involve speeding if the driver is charged with a speeding-related offense or if an officer indicated that racing, driving too fast for conditions, or exceeding the posted speed limit was a contributing factor in the crash. In 2002, 42 percent of intoxicated drivers (i.e., those with BACs of 0.08 percent or higher) in fatal crashes were speeding, as were 43 percent of drivers with BACs of 0.15 percent or higher. In contrast, 15 percent of zero-BAC drivers in fatal crashes were speeding (NHTSA 2003*a*,*d*).

### Safety Belt Use

Drivers who operate motor vehicles after drinking are less likely than other drivers to wear seat belts (see table 7). People who wear safety belts reduce their risk of injury or death in traffic crashes by one-half (NHTSA 2002). At every BAC, a greater percentage of drivers in fatal crashes who survived the crash were wearing seat belts compared with drivers who died in the crash. The higher the driver’s BAC, the less likely he or she was to be wearing a seat belt (NHTSA 2003*a*).

Forty-nine States have laws requiring front seat motor vehicle occupants to wear safety belts. In 31 States, police can only give citations for failure to wear safety belts if a vehicle is stopped for another moving violation (i.e., secondary enforcement). Eighteen States have primary enforcement laws that allow police to stop vehicles and give citations when a motor vehicle occupant is not belted. On average, at least 11 percent more motorists wear safety belts in States with primary safety belt laws (i.e., 80 percent compared with 69 percent in other States) (NHTSA 2003*c*). One study in California found that when the State changed from a secondary to a primary law, the largest percentage increases in safety belt use, nearly 40 percent, were among motorists who were driving after drinking (Lange and Voas 1998). This indicates primary enforcement safety belt laws can be particularly effective in reducing motor vehicle occupant deaths involving drinking drivers.

## Alcohol Dependence and Involvement in Alcohol-Related Crashes

An analysis linking FARS data with data from the National Mortality Follow-Back Survey (NMFS) (Baker et al. 2002) explored the issue of alcohol dependence and involvement in alcohol-related crashes. In the NMFS, researchers contacted informants—usually spouses, parents, children, or siblings—for a sample of deceased people (*n* = 22,957); 83 percent of these contacts provided information about the deceased, including alcohol use history.

NHTSA provided data on BAC and previous drinking and driving convictions for 818 of 1,121 fatally injured drivers in the NMFS. Fatally injured drivers with BACs of 0.15 percent or higher, relative to zero-BAC drivers, were much more likely to have been classified by informants as “problem drinkers” (31 percent vs. 1 percent). This classification was based on the definition of problem drinker as “a person who has physical or emotional problems because of drinking; problems with a spouse, family, or friends because of drinking; problems at work or school because of drinking, or problems with the police because of drinking” (Baker et al. 2002, p. 222). Fatally injured drivers with BACs of 0.15 percent and higher were more likely than those with no BAC to reportedly have five or more drinks at a time at least once per week (43 percent vs. 5 percent), to usually consume five or more drinks on drinking occasions (41 percent vs. 6 percent), to drive after drinking at least once per week (40 percent vs. 4 percent), and to consume five or more drinks when they drank before driving (26 percent vs. 2 percent). Finally, fatally injured drivers with BACs of 0.15 percent or higher were much more likely than those with no BAC to be driving from bars (26 percent vs. 0 percent) or from restaurants or other people’s homes (34 percent vs. 22 percent).

Compared with fatally injured drivers with BACs of 0.10 to 0.14 percent, those with BACs of 0.15 percent and higher were only slightly more likely to to have five or more drinks on drinking days (41 percent vs. 39 percent) or to have five or more drinks on an occasion at least once per week (43 percent vs. 38 percent). However, fatally injured drivers with BACs of 0.15 percent and higher were twice as likely to drive after drinking at least weekly (40 percent vs. 20 percent), and three times more likely to be rated as a problem drinker (31 percent vs. 10 percent).

Evidence about the relationship between alcohol dependence and alcohol-related crashes is also available from the National Longitudinal Alcohol Epidemiologic Survey (NLAES). This 1992 national survey of adults age 18 and older used the Alcohol Use Disorders and Associated Disabilities Interview Schedule (AUDADIS) (Grant and Hasin 1992) to determine whether respondents could be diagnosed with alcohol dependence or alcohol abuse, based on criteria from the *Diagnostic and Statistical Manual, Fourth Edition* (DSM–IV) (American Psychiatric Association [APA] 1994).

Thirteen percent of the respondents were diagnosed as having been alcohol dependent at some point in their lives. This group represented 65 percent of those who had ever been in a motor vehicle crash because of having too much to drink (based on self-report) and 72 percent of those who had been in alcohol-related crashes during the year prior to the interview. Clearly, people who meet established alcohol dependence criteria are disproportionately involved in alcohol-related motor vehicle crashes, accounting for approximately two-thirds of motor vehicle crashes involving alcohol (Hingson et al. 2002).

### Alcoholism Treatment for Drinking and Driving Offenders

Although problem drinkers and people with alcohol dependence account for a large portion of alcohol-related crashes including fatal crashes, currently only 32 States have laws that require people convicted of drinking and driving to be assessed for alcohol abuse or dependence and ordered to attend alcoholism treatment (Mothers Against Drunk Driving [MADD] 2002). A meta-analysis (Wells-Parker et al. 1995) of 215 independent evaluations of mandated treatment of convicted drinking and driving offenders revealed that treatment reduces the incidence of repeat offenses up to 9 percent more than standard sanctions such as license suspensions, revocations, or fines. The average recidivism rate among those who did not receive treatment was 19 percent over a 2-year period. Treatment strategies that combined punishment, education, and therapy with followup monitoring and aftercare were more effective than any single approach for first-time and repeat offenders (Wells-Parker et al. 1995).

Because most drivers in alcohol-related fatal crashes have not recently been convicted for drinking and driving, efforts to screen, diagnose, and treat alcohol problems outside the criminal justice system are also needed. A systematic review of randomized controlled trials to reduce alcohol dependence and abuse among the general population (Dinh-Zarr et al. 1999) has found beneficial effects in reducing not only alcohol consumption but also drinking and driving offenses. Trauma center and emergency department experimental studies of screening and brief intervention counseling for alcohol problems (i.e., very short sessions designed to motivate people to cut down or stop drinking) among people who experienced alcohol-related injuries have also shown reductions in drinking and driving offenses and alcohol-related injuries (Gentilello et al. 1999; Monti et al. 1999; Longabaugh et al. 2001).

## Self-Reported Drinking and Driving

In 1999, researchers conducted a nationwide, random telephone survey of 5,733 adults age 16 and older to collect information about drinking and driving behavior and attitudes, and enforcement of drinking and driving laws (Royal 2000).

Overall, 21 percent of the driving-age public reported driving a vehicle within 2 hours of consuming alcoholic beverages in the previous year, and about 10 percent of these trips were driven at a BAC of 0.08 percent or higher. (BAC was estimated based on a NHTSA formula using gender, weight, number of drinks consumed, length of time drinking, and length of time between last drink and driving.) Bars, taverns, and restaurants were the origin of more than half the drinking and driving trips (54 percent), whereas 17 percent of the trips originated at the driver’s own home and 23 percent originated at another person’s home. In general, people who drive after drinking believe they can consume up to three drinks in a 2-hour period and still drive safely. Those who do not drink and drive think their limit is about one-third less, or two drinks (Royal 2000).

Consistent with the fatality data, males were much more likely to report driving after drinking than females (31 percent vs. 13 percent). Drivers under 21, and particularly those between 16 and 18, were the least likely of any age group of drivers to report driving after drinking, and drivers ages 21 to 45 were the most likely to report this behavior.

Among people who drove after drinking, males reported more drinking–driving trips in the past month than females (an average of 13.2 trips vs. 6.6 trips). Drivers ages 16 to 20 who drove after drinking reported on average the fewest drinking–driving trips of any age group (Royal 2000).

According to survey responses, researchers estimated that in the year prior to the survey, people between the ages of 16 and 20 drove after drinking 12 million times; those between 21 and 29 did so 201 million times; those between 30 and 45, 316 million times; those between 46 and 64, 255 million times; and those 65 and older, 72 million times (Royal 2000).

Not only are males more likely than females to report driving after drinking, they typically drive longer distances after drinking. The average distance of the average drinking–driving trip was greater than 16.7 miles for males, compared with 8.5 miles for females.

Although a smaller percentage of 16- to 20-year-old drivers drive after drinking compared with older drivers, when they do so, 16- to 20-year-olds consume more alcohol before driving. Based on NHTSA’s estimates of the BACs of drivers’ most recent drinking–driving trips (derived from survey results), 16- to 20-year-old drivers had an average BAC of 0.10 percent, three times the average BAC of adults (including 16- to 20-year-old drivers) who drove after drinking. Compounding the danger of driving with higher BACs, drivers ages 16 to 20 on average have 1.4 passengers with them when they drive after drinking, compared with an average of 0.79 passengers for all other age groups. Young drivers’ perceptions about how much they can drink and still drive safely also increase their risk. Among 16- to 20-year-olds, the average BAC at which these drivers considered themselves safe to drive was 0.12 percent for males and 0.07 percent for females (based on respondents’ estimates of how much they could drink in a 2-hour period and still drive safely). In comparison, a 0.05-percent BAC was considered safe by males and females ages 21 to 45, and a 0.03-percent BAC was believed safe by those age 45 and older.

When asked whether they believed their BAC at the time of their most recent drinking–driving trip was above or below the legal limit, about 10 percent of all age drivers believed they were above the legal limit. Forty-four percent of drivers ages 16 to 20 believed they were above the legal limit. This suggests that more than half (56 percent) of the 16- to 20-year-old drinking drivers questioned were not aware that driving after any drinking is illegal for them.

## Drinking and Driving Law Enforcement

The survey data presented above, as well as the fatality and crash statistics outlined earlier, establish the high prevalence of drinking and driving in the United States. Arrests for drinking and driving, by contrast, are less common. There are more than 82 million drinking–driving trips in a given year at BACs of 0.08 and higher (i.e., 10 percent of drinking–driving trips). Although 5 percent of drinking drivers reported driving at 0.08-percent BAC at least once, only 3 percent of drinking drivers were arrested for driving under the influence in the previous year. Between 1978 and 1983, drinking and driving arrests nationwide increased dramatically, from 1.3 million to 1.9 million, but the number of arrests dropped to 1.5 million in 1996 (National Institute on Alcohol Abuse and Alcoholism [NIAAA] 2000) and dropped to 1.4 million in 2001 (NHTSA 2003*b*) Research has shown that publicized sobriety checkpoint programs can reduce alcohol-related traffic crashes and fatalities (Castle et al. 1995; Lacey et al. 1999; Shults et al. 2001). One-third of all drivers reported seeing a sobriety checkpoint in the past year, and 14 percent reported having been in a sobriety checkpoint in the past year (Royal 2000). Also, comprehensive community interventions that include highly publicized enforcement of laws against underage drinking and against drinking and driving have yielded substantial reductions in driving after drinking and in alcohol-related crashes and deaths (Hingson et al. 1996; Holder et al. 2000). (See the sidebar on p. 76 for more information on what can be done to prevent drinking and driving.)

**Reducing Drinking and Driving**

Research has shown that many countermeasures effectively reduce drinking and driving and alcohol-related injuries and deaths (see Hingson et al. 1999 for a review on this topic), including the following proven strategies:

- Greater enforcement of 21 as the minimum legal drinking age, as mandated by the law in all States (Wagenaar et al. 2000; Holder et al. 2000).
- Educating the public about the laws in effect in all States that make it illegal for people under 21 to drive after drinking (Blomberg 1992).
- Enactment and enforcement of laws making it a criminal offense per se to drive above the legal blood alcohol concentration (BAC), which are in effect in all 50 States (Voas et al. 2000).
- Enactment and enforcement of administrative license revocation (ALR) laws, in place in 40 States, which allow police to immediately seize the driver’s license of drivers operating above the legal BAC (Zador et al. 1989; Voas et al. 2000). ALR laws have been associated with greater deterrence than criminal per se laws, as measured by alcohol-related fatal crash reductions, because ALR produces immediate (more swift and certain) license revocation.
- Enactment and enforcement of primary enforcement safety belt laws, in effect in 18 States, which permit police to cite motorists for not wearing safety belts (Dinh-Zarr et al. 2001).
- Mandatory alcohol assessment and treatment of offenders convicted for drinking and driving, which is the law in 32 States (Wells-Parker et al. 1995).
- Enactment and enforcement of 0.08-percent legal BAC limits for adult drivers, in effect in 44 States (Shults et al. 2001; Voas et al. 2000; Hingson et al. 1999, 2000).
- Lowering legal BAC limits for convicted drinking and driving offenders, as three States have done (Hingson et al. 1998).
- Impounding vehicles or license plates of repeat offenders (Voas et al. 1997*a*,*b*, 1998).
- Mandating the use of ignition interlocks for people convicted of drinking and driving (Beck et al. 1999).

Publicized sobriety checkpoints (Castle et al. 1995; Lacey et al. 1999; Shults et al. 2001) and comprehensive community education and enforcement programs (Hingson et al. 1996; Holder et al. 2000) have also been found to reduce alcohol-related fatal and injury crashes.

If all States adopted these countermeasures, which have been shown to reduce alcohol-related motor vehicle crash injuries and deaths, it is possible the United States would again experience declines in alcohol-related deaths and injuries.

—Ralph Hingson and Michael Winter

ReferencesAmerican Psychiatric Association (APA)Diagnostic and Statistical Manual of Mental DisordersFourth EditionWashington, DCAPA1994GrantBThe impact of family history of alcoholism on the relationship between age of onset of alcohol use and DSM–III alcohol dependenceAlcohol Health & Research World222144147199815706789PMC6761809GrantBFHasinPSThe Alcohol Use Disorders and Associated Disabilities Interview ScheduleRockville, MDNational Institute on Alcohol Abuse and Alcoholism1992HingsonRHeerenTJamankaAHowlandJAge of drinking onset and unintentional injury involvementJAMA: Journal of the American Medical Association284121527153320001100064610.1001/jama.284.12.1527HingsonRHeerenTLevensonSAge of drinking onset, driving after drinking and involvement in alcohol-related motor vehicle crashesAccident Analysis and Prevention34859220021178957810.1016/s0001-4575(01)00002-1O’MalleyPMWagenaarACEffects of minimum drinking age laws on alcohol use, related behavior, and traffic crash involvement among American youth: 1976–1987Journal of Studies on Alcohol5254784911991194310510.15288/jsa.1991.52.478ShultsRElderRSleetDReviews of evidence regarding interventions to reduce alcohol-impaired drivingAmerican Journal of Preventive Medicine21Suppl 46688200110.1016/s0749-3797(01)00381-611691562

The NHTSA survey described above (Royal 2000) also asked participants about their perceived chance of being stopped and arrested for drinking and driving. More than half the respondents thought it would be at least somewhat likely that they would be stopped by the police if they drove after having too much to drink. However, 38 percent of respondents believed it would be at least somewhat likely that if they drove after drinking too much they would be stopped by the police, arrested, and convicted. Only 2 percent believed it would be almost certain that all three of these things would happen.

## Trends in Drinking and Driving

This section examines trends in drinking and driving over approximately the past 20 years. Trends are reported based both on surveys of drivers stopped at random while driving and on records of alcohol-related fatal crashes.

### National Roadside Surveys

National roadside surveys were conducted in 1973, 1986, and 1996. Drivers were stopped between 10:00 p.m. and 3:00 a.m. on Friday and Saturday nights, when most drinking occurs (Voas et al. 1997). Similar sites and sampling procedures were used in each survey.

Overall, the proportion of drivers with positive BACs decreased from 36 percent in 1973 to 17 percent in 1996. The decline was greatest for drivers with lower BACs (0.005 to 0.049 percent).

The percentage of drivers under age 21 who had BACs of 0.10 or higher fell from 4.1 to 0.3 percent, representing the greatest proportional decline for any age group. Among 21- to 25-year-olds, the proportion of drivers with BACs of 0.10 percent or higher decreased from 5.7 to 3.8 percent.

The percentage of drivers with BACs of 0.10 percent or higher declined from 3.0 to 1.5 percent among females and from 5.5 percent to 3.5 percent among males. BAC levels also varied by race/ethnicity. Among White drivers, the proportion with positive BACs declined from 5.1 to 2.3 percent. Among African American drivers, the proportion with positive BACs declined from 6.0 to 3.6 percent. By contrast, the proportion of Hispanic drivers with positive BACs increased from 3.3 to 7.5 percent. The number of Hispanic drivers surveyed increased sevenfold during that time period (Voas et al. 1997).

### Traffic Fatality Trends

Consistent with the roadside survey results, traffic deaths involving alcohol declined markedly from the early 1980s to 1996, but during the last 6 years the downward progress has abated and alcohol-related traffic deaths have actually increased somewhat (NHTSA 2003*a*).

**Figure 1:**
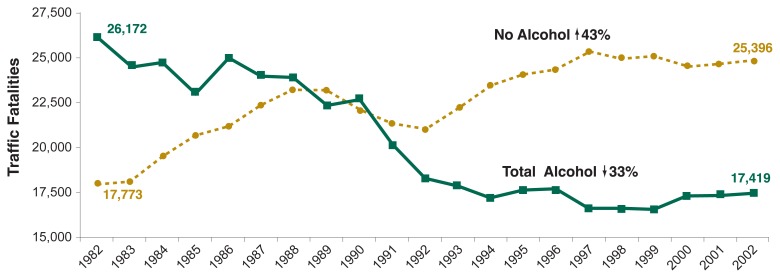
Trends in alcohol-related and non-alcohol-related traffic fatalities, 1982 through 2002. Alcohol-related traffic deaths were 33 percent lower in 2002 than in 1982. During the same time, traffic deaths that did not involve alcohol increased 43 percent.

As shown in figure 1, in 1982 when NHTSA first made nationwide estimates, there were 26,172 alcohol-related traffic deaths. Alcohol-related traffic deaths were 33 percent lower in 2002, at 17,419. During the same timeframe, traffic deaths that did not involve alcohol increased 43 percent, from 17,773 to 25,396. For every 100 million vehicle miles traveled, the rate of both non-alcohol-related and alcohol-related traffic deaths declined (19 percent and 62 percent, respectively). This is attributable to a 77-percent increase in the number of miles driven in the United States, from 1,595 billion in 1982 to 2,830 billion in 2002.

**Figure 2:**
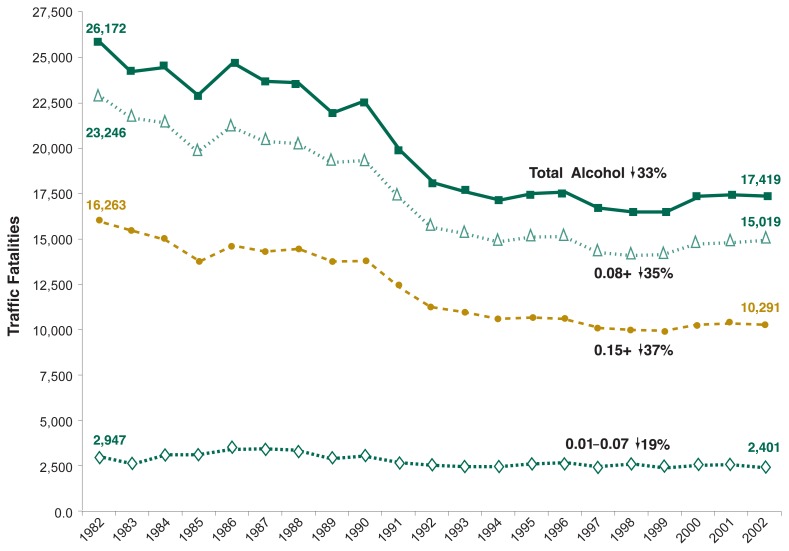
Trends in number of alcohol-related traffic fatalities for different BACs, 1982 through 2002. Traffic deaths involving people with BACs up to 0.08 percent had the smallest proportional decline (19 percent) from 1982 through 2002. Traffic deaths among people with BACs of 0.08 percent and higher declined 35 percent, and those involving people with BACs of 0.15 percent and higher declined 37 percent. SOURCE: NHTSA 2003*a*.

Although total alcohol-related traffic fatalities have decreased, the degree of decline varies when BAC is considered. The numbers of traffic deaths involving people with BACs up to 0.08 percent had the smallest proportional decline (19 percent) from 1982 to 2002 (see figure 2). Traffic deaths among people with BACs of 0.08 percent and higher declined 35 percent, and those involving people with BACs of 0.15 percent and higher declined 37 percent.

Declines in traffic deaths since 1982 have not varied much by gender. In 1982, 20,365 men and 5,805 women died in alcohol-related crashes. In 2002, the number of male alcohol-related traffic deaths was 13,500, a 34-percent decline. Among women, 3,910 deaths were recorded in 2002, a 33-percent reduction. However, there have been greater proportional declines in the numbers of male drivers in alcohol-related fatal crashes. Since 1982 the number of male drivers in alcohol-related fatal crashes declined 37 percent, from 19,478 to 12,270, whereas the number of female drivers only declined 22 percent, from 2,854 to 2,216.

**Figure 3:**
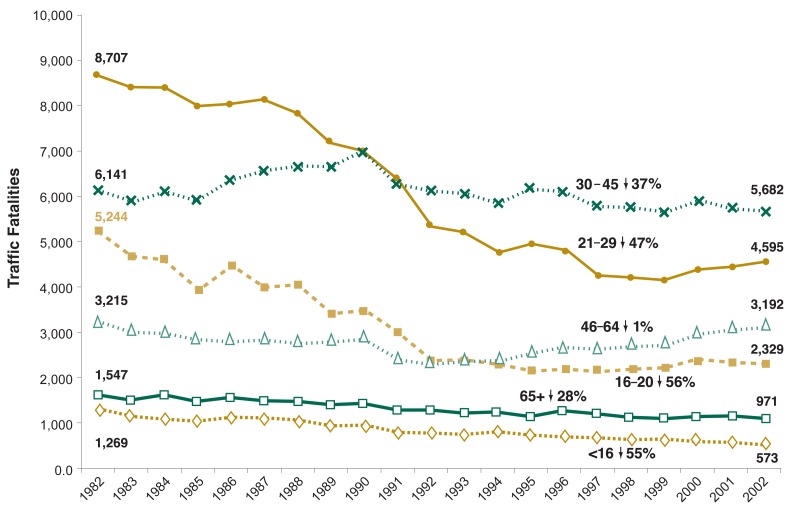
Trends in number of alcohol-related traffic fatalities for different age groups, 1982 through 2002. Sixteen- to 20-year-olds have had the greatest decline in alcohol-related traffic deaths since 1982, down 56 percent. Alcohol-related traffic deaths declined 47 percent among 21- to 29-year-olds and 37 percent among 30- to 45-year-olds. The smallest proportional decline was observed among 46- to 64-year-olds, where only a 1-percent reduction occurred. SOURCE: NHTSA 2003*a*.

Young adults have experienced a greater proportional reduction in alcohol-related traffic deaths than older adults in the last 20 years. Sixteen- to 20-year-olds have had the greatest decline in alcohol-related traffic deaths since 1982, down 56 percent, from 5,244 to 2,329 (see figure 3). There was a 62-percent decline in traffic deaths among young people in which the person with the highest BAC in the crash had a BAC above 0.15 percent, and a 59-percent decline in deaths where BACs exceeded 0.08 percent.

Alcohol-related traffic deaths declined 47 percent among 21- to- 29-year-olds (from 8,707 to 4,595) and 7 percent among 30- to 45-year-olds (from 6,141 to 5,682).The smallest proportional decline was observed among 46- to 64-year-olds, where only a 1-percent reduction occurred, from 3,215 to 3,192 (NHTSA 2003*a*).

Over the past two decades, fatal crashes not involving alcohol increased in each age group, indicating that the overall decline in alcohol-related deaths during this period was independent of changes in the age composition of the U.S. population.

The greater decline in alcohol-related traffic deaths among 16- to 20-year-olds is in part attributable to the adoption of age 21 as the legal drinking age, which occurred in all States by 1988. A review of more than 49 studies of changes in the legal drinking age revealed that in the 1980s and 1990s when many States lowered the legal drinking age, alcohol-related traffic crashes involving drivers under 21 increased 10 percent. In contrast, when States increased the legal drinking age to 21, alcohol-related crashes among people under 21 decreased an average of 16 percent (Shults et al. 2001).

Zero tolerance laws have also contributed to these declines. These laws, which have been enacted in every State, make it illegal for drivers under 21 to drive after any drinking. A comparison of the first eight States to adopt zero tolerance laws with nearby States without these laws revealed a 21-percent decline in the proportion of fatal crashes among drivers under 21 that were of the type most likely to involve alcohol (i.e., single-vehicle fatal crashes at night) (Hingson et al. 1994). This type of crash among adults (i.e., those age 21 and older) declined 3 to 4 percent both in zero tolerance States and comparison States. Wagenaar and colleagues (2000) found that in the first 30 States to adopt zero tolerance laws, relative to the rest of the nation, there was a 19-percent decline in the proportion of people under 21 who drove after any drinking, and a 23-percent decline in the proportion of those driving after five or more drinks (based on survey results).

## Summary

Despite marked reductions in the proportions of motorists who drive after drinking and in alcohol-related traffic fatalities, alcohol-impaired driving remains a serious threat to the nation’s health. In 2002, 41 percent of traffic deaths and 9 percent of traffic injuries were alcohol related. As many as 44 percent of people killed in crashes involving drinking drivers are people other than the drinking driver.

Fatal crash risk was at least 11 times higher for drivers with BACs of 0.08 percent, the legal limit for intoxication in most States, than for drivers with zero BACs. Fatal crash risk was 52 times higher for male drivers ages 16 to 20 with BACs of 0.08 percent, compared with zero-BAC drivers of the same age. Fatal crash risk nearly doubled with each 0.02-percent increase in BAC.

Traffic deaths are most likely to be alcohol related among males, Native Americans and Mexican Americans, people ages 21 to 45, those who die in motor vehicle crashes on weekend nights, and people with symptoms of alcohol dependence.

National surveys also reveal that males and people ages 21 to 45 are the most likely to drive after drinking. Although drivers ages 16 to 20 accounted for only 12 million of 957 million drinking–driving trips in 1999, the average BAC among young drinking drivers was 0.10 percent, more than three times the average BAC level for all drinking drivers. Drivers under 21 in all States tend to drive with more passengers in their vehicles. Despite laws making it illegal to sell alcohol to people under 21 and for drivers that age to drive after any drinking, most people in this age group who drive after drinking are unaware that it is illegal to do so.

Recent evidence indicates that the younger people are when they begin drinking, the greater their likelihood of becoming alcohol dependent, which may increase their risk of alcohol-related crash involvement not only during adolescence but during adulthood as well (see the sidebar on p. 75).

**Age of Drinking Onset and Alcohol-Related Crash Involvement**

Evidence about the relationship between age of drinking onset and alcohol-related crash involvement is available from the National Longitudinal Alcohol Epidemiologic Survey (NLAES). This 1992 national survey of adults age 18 and older used the Alcohol Use Disorders and Associated Disabilities Interview Schedule (AUDADIS) (Grant and Hasin 1992) to determine whether respondents could be diagnosed with alcohol dependence or alcohol abuse, based on criteria from the *Diagnostic and Statistical Manual, Fourth Edition* (DSM–IV) (APA 1994).

NLAES data showed that the younger respondents were when they first began to drink alcohol, the more likely they were to develop alcohol dependence (Grant 1998). Among both males and females and people with and without a family history of alcoholism, those who began drinking regularly before age 14 were at least three times more likely to develop alcohol dependence during their lives than those who waited until age 21 or older to drink (Grant 1998).

Further analyses of that survey revealed that even after controlling for history of alcohol dependence, those who started drinking at younger ages were more likely to drink heavily (five or more drinks per occasion) with greater frequency (Hingson et al. 2000). Moreover, the younger people were when they began drinking, the greater their likelihood of driving after drinking too much and of being in motor vehicle crashes because of drinking (based on self-report). Compared with respondents who waited until age 21 or older to start drinking, those who began drinking before age 14 were three times more likely to report ever driving after drinking too much (58 percent vs. 18 percent) and four times more likely to report doing so in the year prior to the survey (13 percent vs. 3 percent). Those who started drinking before age 14 were seven times more likely to have been in a drinking-related motor vehicle crash at any time in their lives (14 percent vs. 2 percent) and in the past year (0.7 percent vs. 0.1 percent) (Hingson et al. 2002). Because the average age of respondents in the survey was 44, these findings indicate that those who start drinking at an early age are more likely to be in alcohol-related motor vehicle crashes during both adolescence and adulthood.

It is noteworthy that this pattern of relationships was found even after controlling for history of alcohol dependence, frequency of heavy drinking, years of drinking, age, gender, race/ethnicity, education, marital status, smoking, and illicit drug use, all of which were related to the age at which respondents began to drink.

Research has long indicated that raising the minimum legal drinking age (MLDA) to 21 has reduced alcohol-related crashes among drivers under 21 (Shults et al. 2001). This study raises the possibility that delaying underage drinking may reduce alcohol-related crash involvement among adults as well (Hingson et al. 2002). In an analysis of the effects of increasing the MLDA to 21, O’Malley and Wagenaar (1991) found that people who grew up in States with the legal drinking age of 21 not only drank less when they were younger than 21, they also drank less from ages 21 to 25.

—Ralph Hingson and Michael Winter

ReferencesBeckKHRoochWJBakerEAEffects of the ignition interlock license restrictions on drivers with multiple offenses: A randomized trial in MarylandAmerican Journal of Public Health89111690170019991055339110.2105/ajph.89.11.1696PMC1508998BlombergRDLower Legal BAC Limits for Youth: Evaluation of the Maryland 0 .02 LawPub. No. DOT HS–806–807Washington, DCU.S. Department of Transportation1992CastleSPThompsonJDSpataroJAEarly Evaluation of a Statewide Sobriety Checkpoint ProgramPaper presented at the 39th Annual Conference of the Association for the Advancement of Automotive MedicineChicagoOctober 16–18, 1995Dinh-ZarrTBSleetDAShultsRAReviews of evidence regarding interventions to increase the use of safety beltsAmerican Journal of Preventive Medicine2Suppl 44865200110.1016/s0749-3797(01)00378-611691561HingsonRMcGovernTHowlandTHeerenTReducing alcohol-impaired driving in Massachusetts: The Saving Lives ProgramAmerican Journal of Public Health867917971996865965110.2105/ajph.86.6.791PMC1380396HingsonRHeerenTWinterMEffects of Maine’s 0.05 percent legal blood alcohol level for drivers with DWI convictionsPublic Health Reports11344044619989769769PMC1308415HingsonRHeerenTWinterMPreventing impaired drivingAlcohol Research & Health2313139199910890796PMC6761696HingsonRHeerenTWinterMEffects of recent 0.08 percent legal blood alcohol limits on fatal crash involvementInjury Prevention610911420001087566610.1136/ip.6.2.109PMC1730627HolderHDGruenewaldPJPonietioWREffect of community-based interventions on high-risk drinking and alcohol-related injuriesJAMA: Journal of the American Medical Association284182341234720001106618410.1001/jama.284.18.2341LaceyJHJonesRKSmithRGEvaluation of Checkpoint Tennessee: Tennessee’s Statewide Sobriety Checkpoint ProgramPub. No. DOT HS–808–841Washington, DCU.S. Department of Transportation, National Highway Traffic Safety Administration1999ShultsRElderRSleetDReviews of evidence regarding interventions to reduce alcohol-impaired drivingAmerican Journal of Preventive Medicine21Suppl 46688200110.1016/s0749-3797(01)00381-611691562VoasRBTippettsALangeJEvaluation of a method for reducing unlicensed driving: The Washington and Oregon license plate sticker lawsAccident Analysis and Prevention2956276341997a931671010.1016/s0001-4575(97)00014-6VoasRBTippettsATaylorETemporary vehicle immobilization: Evaluation of a program in OhioAccident Analysis and Prevention2956356421997b931671110.1016/s0001-4575(97)00016-xVoasRBTippettsATaylorETemporary vehicle impoundment in Ohio: A replication and confirmationAccident Analysis and Prevention3056516561998967821810.1016/s0001-4575(98)00008-6VoasRTippettsAFellJThe relationship of alcohol safety laws to drinking drivers in fatal crashesAccident Analysis and Prevention3248349220001086875110.1016/s0001-4575(99)00063-9WagenaarACMurrayDMGebanJPCommunities Mobilizing for Change: Outcomes from a randomized community trialJournal of Studies on Alcohol611859420001062710110.15288/jsa.2000.61.85Wells-ParkerEBangert-DrownsRMcmillenRWilliamsMFinal results from a meta-analysis of remedial interventions with drink/drive offendersAddiction909079261995766331310.1046/j.1360-0443.1995.9079074.xZadorPLundAFieldsMWeinbergKFatal crash involvement and laws against alcohol-impaired drivingJournal of Public Health Policies10446748519892621251

Drinking drivers are less likely to wear safety belts, and the higher the BAC of a fatally injured driver, the less likely he or she was to have been wearing a safety belt. Failure to wear safety belts increases the risk of injury or death in fatal crashes.

Despite overall marked reductions in alcohol-related traffic deaths since the early 1980s, there has been little reduction since the mid-1990s, and alcohol-related traffic deaths have increased slightly in the past 3 years. Proportional reductions in alcohol-related traffic deaths were smaller among people with prior drinking and driving offenses than among those without previously recorded offenses.

Nationally, the number of arrests for drinking and driving increased sharply from the late 1970s to the early 1980s, but were substantially lower in the 1990s.

A national survey conducted in 1999 revealed that only 30 percent of adults age 16 and older believed it at least somewhat likely that if they drove after drinking too much they would be stopped by the police, arrested, and convicted. Only 2 percent believed it almost certain that all those things would happen. In 1999, according to that survey, 82 million (or 10 percent) of drinking and driving trips involved a driver with a BAC of 0.08 percent or higher. However, only 1.5 million drivers were arrested for driving under the influence of alcohol or drugs that year.

## Figures and Tables

**Table 1:** Traffic Deaths, by Age and BAC, 2002

	Age						

	<16	16–20	21–29	30–45	46–64	65+	unknown
BAC*							
0.00%	77%	63%	43%	47%	62%	85%	51%
0.01–0.07%	5%	7%	7%	6%	5%	3%	5%
0.08–0.14%	7%	13%	16%	13%	9%	5%	16%
0.15% +	11%	17%	34%	35%	24%	7%	29%
Percentage alcohol-involved	23	37	57	53	38	15	49
Number alcohol-involved	573	2,329	4,595	5,682	3,192	971	78
Total fatalities	2,542	6,277	8,022	10,707	8,487	6,622	158

* BAC = the highest blood alcohol concentration of a driver or pedestrian involved in the crash.

NOTE: Alcohol-related traffic deaths are more likely to occur at lower BACs among 16- to 20-year-olds, compared with other age groups. Only 17 percent of alcohol-related traffic fatalities in this age group occurred at BACs of 0.15 percent or higher (i.e., a driver or pedestrian involved in the crash had a BAC of at least 0.15 percent). Among 21- to 29-year-olds, 34 percent of alcohol-related fatal crashes occurred at BACs of at least 0.15 percent.

SOURCE: NHTSA 2003*a*.

**Table 2:** Traffic Crashes, by Time of Day and BAC, 2002 (in percent)

	BAC*				

	0.00% (*n* = 22,683)	0.01–0.07% (*n* = 2,129)	0.08–0.14% (*n* = 4,204)	0.15%+ (*n* = 9,293)	0.01%+ (*n* = 15,626)
6:00 p.m. – midnight	23	38	38	41	40
Midnight – 6:00 a.m.	10	29	37	39	37
6:00 a.m. – noon	28	11	8	6	7
Noon – 6:00 p.m.	39	21	16	13	15

* BAC = the highest blood alcohol concentration of a driver or pedestrian involved in the crash.

NOTE: Fatal alcohol-related traffic crashes are more likely to occur at night than during the day. For example, 77 percent (40 plus 37) of fatal alcohol-related traffic crashes occurred between 6:00 p.m. and 6:00 a.m., compared with 33 percent (23 plus 10) of non-alcohol-related fatal crashes.

SOURCE: NHTSA 2003*a*.

**Table 3:** Fatal Crashes by Day of the Week and BAC, 2002 (in percent)

	BAC*				

	0.00% (*n* = 22,683)	0.01–0.07% (*n* = 2,129)	0.08–0.14% (*n* = 4,204)	0.15%+ (*n* = 9,293)	0.01%+ (*n* = 15,626)
Monday	14	10	9	9	9
Tuesday	14	11	9	9	9
Wednesday	15	10	11	9	10
Thursday	14	12	12	11	11
Friday	16	17	16	15	16
Saturday	14	22	24	25	24
Sunday	13	18	20	21	21

* BAC = the highest blood alcohol concentration of a driver or pedestrian involved in the crash.

NOTE: For example, 14 percent of all non-alcohol-related (zero-BAC) fatal crashes occurred on Mondays. More alcohol-related crashes occur on Saturdays (24 percent) than any other day.

SOURCE: NHTSA 2003*a*.

**Table 4:** Drivers in Fatal Traffic Crashes and Driver’s Record of Drinking and Driving Conviction Within the Last 3 Years, by BAC, in 2002

	No Prior D&D* Conviction	Prior D&D Conviction	Percent of Drivers in Fatal Crashes Who Had Prior D&D Conviction
	
	Number of Drivers in Fatal Crashes/% of Total Drivers in Fatal Crashes		
Driver’s BAC			
0.00%	40,963 (76)	621 (34)	1
0.01–0.07%	2,118 (4)	107 (6)	5
0.08–0.14%	3,789 (7)	289 (16)	7
0.15%+	6,905 (13)	831 (45)	10
Total drivers in fatal crashes with BAC of 0.01%+	12,811 (24)	1,227 (66)	8
Total drivers in fatal crashes	53,774 (100)	1,848 (100)	3

* D&D = drinking and driving.

NOTE: Only a small fraction of drivers in fatal crashes had drinking and driving convictions in the previous 3 years (3.2 percent). But of the drivers in alcohol-related fatal crashes who had BACs pf 0.01 percent and higher, 8.4 percent had prior drinking and driving convictions. Further, the higher the BAC of drivers in fatal crashes, the greater their likelihood of a prior conviction.

SOURCE: NHTSA 2003*a*.

**Table 5:** Driver Behaviors and Characteristics in Fatal Traffic Crashes, by BAC, 2002 (in percent)

	Driver’s BAC			

	0.00% (*n* = 43,141)	0.01–0.07% (*n* = 2,317)	0.08–0.14% (*n* = 4,274)	0.15%+ (*n* = 8,070)
Failure to keep in lane or ran off road	23	43	49	58
Driving too fast for conditions	15	33	38	43
Failure to yield right of way	10	6	5	3
Inattentive	6	7	7	8
Failure to obey traffic signals	5	5	5	5
Operating in a reckless or erratic fashion	4	10	12	12
Overcorrecting	3	5	6	8
Driving wrong way	<1	<1	<1	<1
Operator inexperienced	<1	<1	<1	<1

NOTE: For example, only 23 percent of zero-BAC drivers in fatal crashes failed to keep in their lane or ran off the road, compared with 58 percent of drivers with BACs of 0.15 percent or higher.

SOURCE: NHTSA 2003*a*.

**Table 6:** Most Harmful Event in Fatal Traffic Crashes, by Driver’s BAC, 2002 (in percent)

	Driver’s BAC			
	0.00% (*n* = 43,141)	0.01–0.07% (*n* = 2,317)	0.08–0.14% (*n* = 4,274)	0.15%+ (*n* = 8,070)
Overturned	10	20	24	28
Struck pedestrian	10	7	6	4
Struck moving vehicle	66	44	36	29
Struck fixed object	10	23	28	33
Other	4	6	6	6

NOTE: Among drivers with zero BAC in fatal crashes, only 10 percent experienced a vehicle overturn, and only 10 percent struck a fixed object. In contrast, among drivers with BACs of 0.15 percent or higher, 28 percent experienced a vehicle overturn and 33 percent struck a fixed object.

SOURCE: NHTSA 2003*a*.

**Table 7:** Seat Belt Use by Drivers in Fatal Crashes, by Driver’s BAC, 2002

	Driver’s BAC			

	0.00% *n* (%)	0.01–0.07% *n* (%)	0.08–0.14% *n* (%)	0.15%+ *n* (%)
Drivers in fatal crashes who survived the crash	25,287	986	1,704	1,861
Percent belted	(79)	(56)	(51)	(43)
Fatally injured drivers	14,685	995	1,973	5,412
Percent belted	(48)	(33)	(24)	(20)

NOTE: At every BAC, a greater percentage of drivers who survived fatal crashes were wearing seat belts than were drivers who did not survive. The higher the driver’s BAC, the less likely he or she was to be wearing a seat belt.

SOURCE: NHTSA 2003*a*.
